# Local and regional drivers of ant communities in forest-grassland ecotones in South Brazil: A taxonomic and phylogenetic approach

**DOI:** 10.1371/journal.pone.0215310

**Published:** 2019-04-11

**Authors:** William Dröse, Luciana Regina Podgaiski, Camila Fagundes Dias, Milton de Souza Mendonça

**Affiliations:** 1 Programa de Pós-Graduação em Biologia Animal, Universidade Federal do Rio Grande do Sul, Porto Alegre, Rio Grande do Sul, Brazil; 2 Departamento de Ecologia, Universidade Federal do Rio Grande do Sul, Porto Alegre, Rio Grande do Sul, Brazil; USDA Forest Service, UNITED STATES

## Abstract

Understanding biological community distribution patterns and their drivers across different scales is one of the major goals of community ecology in a rapidly changing world. Considering natural forest-grassland ecotones distributed over the south Brazilian region we investigated how ant communities are assembled locally, i.e. considering different habitats, and regionally, i.e. considering different physiographic regions. We used taxonomic and phylogenetic approaches to investigate diversity patterns and search for environmental/spatial drivers at each scale. We sampled ants using honey and tuna baits in forest and grassland habitats, in ecotones distributed at nine sites in Rio Grande do Sul state, Brazil. Overall, we found 85 ant species belonging to 23 genera and six subfamilies. At the local scale, we found forests and grasslands as equivalent in ant species and evolutionary history diversities, but considerably different in terms of species composition. In forests, the soil surface air temperature predicts foraging ant diversity. In grasslands, while the height of herbaceous vegetation reduces ant diversity, treelet density from forest expansion processes clearly increases it. At a regional scale, we did not find models that sufficiently explained ant taxonomic and phylogenetic diversity based on regional environmental variables. The variance in species composition, but not in evolutionary histories, across physiographic regions is driven by space and historical processes. Our findings unveil important aspects of ant community ecology in natural transition systems, indicating environmental filtering as an important process structuring the communities at the local scale, but mostly spatial processes acting at the regional scale.

## Introduction

Ants are extremely abundant and ecologically important organisms widespread through ecosystems worldwide [1]. Several mechanisms shape ant distribution patterns such as environmental conditions (i.e. that filter species or lineages according to habitat requirements), species interactions, historical and geographical factors (i.e. affecting dispersal) [2]. Indeed, depending on the spatial scale considered ants might show different distribution patterns (e.g. [3,4]). For example, at smaller or local scales, microclimatic variation [5,6], soil and vegetation characteristics [7–9] and interspecific competition [10,11] usually act on community assembly. At broad or regional scales, climate variables [12,13], altitude [14,15], latitude [16–18] and dispersal limitation [19,20] may explain most of the patterns. All these predictors, acting in isolation or interacting, play roles in ant community diversity and distribution patterns of evolutionary lineages [2].

Ecotones are zones where adjacent ecological systems co-occur in space, supporting unique ecological dynamics [21]. Their definition is scale-dependent, including from biomes or ecoregions, to landscape patches or vegetation communities [22]. An example of an ecotone widespread through the globe is the contact between grassland/savannas and forests. Such contrasting habitats differ in relation to several environmental characteristics and conditions, which select adapted species and evolutionary histories from the regional pool [23,24]. While forests may harbor species more associated with deep shade, moisture and buffered temperatures, grasslands, on the contrary, may favor shade-intolerant species and those more prone to microclimatic oscillations [25].

In South Brazil, the current warm and moist climate favors forest expansion processes over native grasslands in many physiographic regions, forming mosaic landscapes [26]. Fire and grazing have potential roles of controlling forest expansion without causing major damage to grasslands, but in ecotones where disturbances are low or even absent, the establishment and growth of shrubs and treelets, which are good light competitors, inhibits typical grassland plant diversity [27]. Here we aim to investigate ant community diversity patterns in this system, and search for potential drivers, considering local (different habitats: forests and grasslands) and regional scales (different physiographic regions over Rio Grande do Sul state).

Diversity patterns and their drivers are often explored in the context of the taxonomic identity of the species. In addition to the description of the taxonomic diversity (TD), the use of the evolutionary history (phylogenetic diversity—PD) and ecological traits (functional diversity—FD) of a community are useful to understand these patterns of distribution in a historical and ecological context. Taking into account that higher evolutionary diversification might result in higher functional diversification, recent studies have suggested that PD can be an effective proxy for FD, particularly in the absence of trait data (e.g. [28]). In the case of ant communities, PD and FD have been highly correlated as reported in many studies [20,29,30], meaning that the traits display phylogenetic signals, i.e. they are evolutionarily conserved. Therefore, PD can be a potentially useful tool to estimate functional diversity in ant communities. On the other hand, TD may not always converge with the patterns of PD and FD [30], e.g. when two communities with equal TD have different levels of functional redundancy and evolutionary histories, and then their information can be complementary. Here we used both TD and PD to explore ant diversity distributions patterns in forest-grassland ecotones. Based on the available literature, we elaborate some predictions.

Locally, we expect compartmented ant assemblages inhabiting forests and grasslands, but no detection of differences regarding species diversity between habitats, as reported similarly by Pinheiro et al. [31] and Klunk et al. [32] for the same region. As ants are thermophilic organisms, local temperature should positively influence the diversity of ant species found foraging [33]. In forests, structural properties such as leaf-litter depth should increase microhabitat complexity and thus support increased ant species diversity [8,34]. In grasslands, suppression or diminished disturbance frequency/intensity (e.g. fire and grazing), as measured by herbaceous vegetation height and shrub density, should reduce ant diversity [35], although tree densification through forest expansion may increase it due to the higher availability of resources [36].

Regionally, we expect distinct ant composition among physiographic regions of Rio Grande do Sul state [37], and since ants lack in efficient large-scale dispersal mechanisms [19,20], both environmental and spatial factors may contribute to this variation. Based on the water-energy dynamics hypothesis [38], regional temperature and precipitation patterns should positively affect ant diversity [12,13,18,39], while altitude affects it negatively [14,15].

## Materials and methods

### Ethics statement

Permission to carry out this study in private lands was granted by landowners; sites within conservation units had authorization granted by the Environmental Secretariat of Rio Grande do Sul state (SEMA, Brazil).

### Study area and design

**Fig 1:**
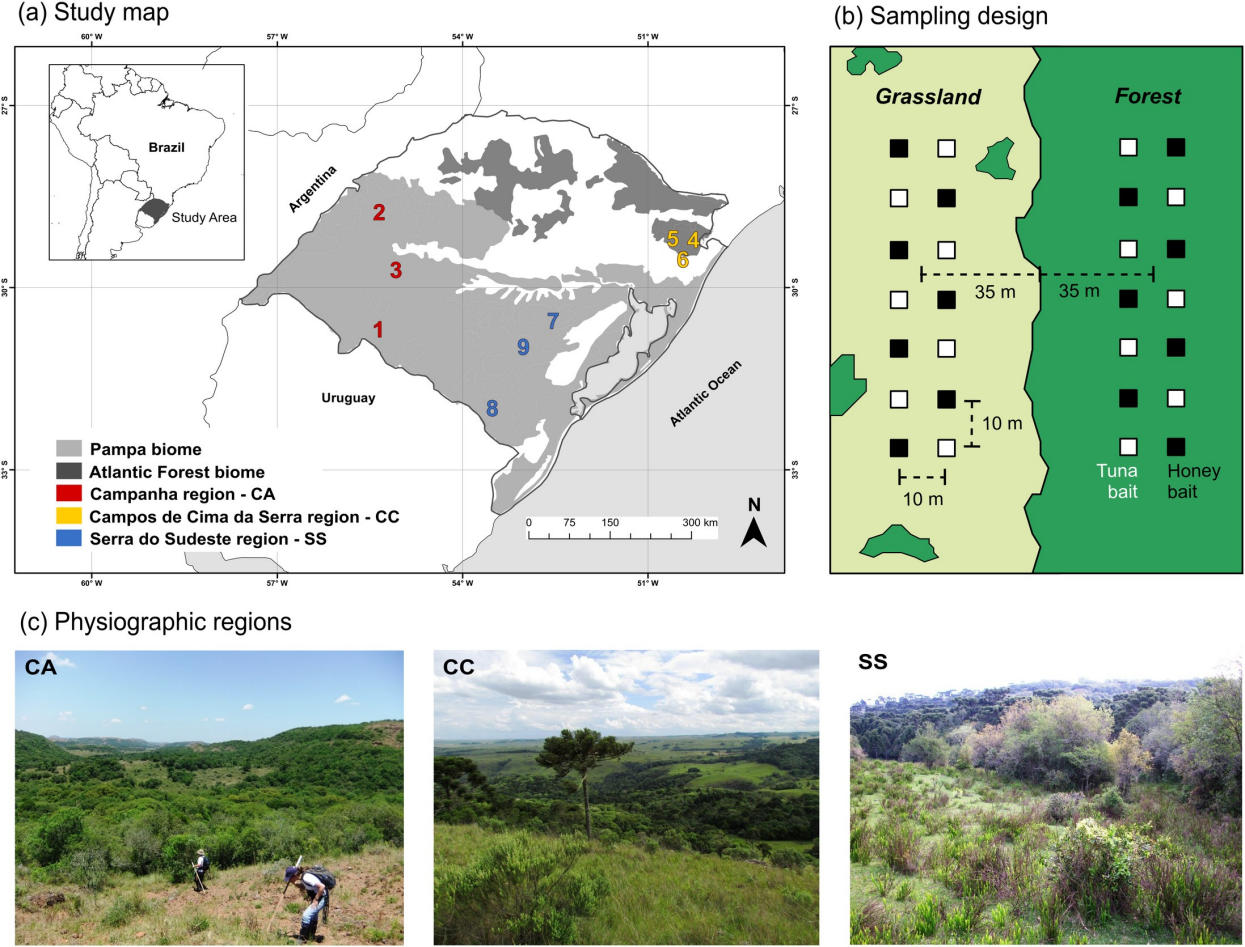
Map of the study sites and types of forest-grassland ecotones from different physiographic regions. (a) Forest-grassland ecotones sampled in nine localities belonging to three different physiographic regions of Rio Grande do Sul state, Brazil: Campanha region (red numbers: 1-Santana do Livramento; 2-Santo Antônio das Missões; 3-São Francisco de Assis), Campos de Cima da Serra region (yellow numbers: 4-Cambará do Sul; 5-Jaquirana; 6-São Francisco de Paula) and Serra do Sudeste region (blue numbers: 7-Encruzilhada do Sul; 8-Herval; 9-Santana da Boa Vista). (b) Sampling design with bait points. (c) Different physiognomies of forest-grassland ecotones sampled.

We studied forest-grassland ecotones along nine sites in Rio Grande do Sul state, Brazil. Grasslands occur on areas in both the Atlantic Forest and Pampa biomes and form mosaic landscapes with forests [40]. The sites sampled belong to three different physiographic regions: (i) Campanha, (ii) Campos de Cima da Serra and (iii) Serra do Sudeste (henceforth CA, CC and SS, respectively) (Fig 1A). The nearest sites were about 36 km apart (Cambará do Sul and Jaquirana municipalities) and the most distant sites (Cambará do Sul and Santana do Livramento municipalities) were about 553 km apart. The physiographic regions differ in terms of climate, vegetation, soil types and biotic evolutionary histories. The climate is Cfb type in the CA and SS regions, according to the Köppen climate classification. Only CC and the high altitude sites in SS are classified as Cfa type climate [41]. There is an environmental temperature gradient across the three regions, decreasing from southwest to northeast (i.e. from CA to CC) (see S3 Table). Mean altitude in sites sampled at CA is 185 m, while at CC it is 883 m and at SS 240 m.

Each studied site was delimited by a 2 x 2 km grid where we selected two forest-grassland ecotones at least 1 km from each other. The sole exception was one site from the CC region, where we studied only one ecotone (totaling 17 ecotones). Ecotones from each physiographic region are formed by different natural grassland types associated with specific forest remnants: CA region–Deciduous Seasonal Forest and Sand and Soil Shallow Grasslands; CC region–Mixed Ombrophilous Forest and Highland Grasslands; and SS region–Semideciduous Seasonal Forest and Shrub Grasslands [40,42] (Fig 1C). At each ecotone, we sampled ant communities in both forests and grasslands.

### Ant sampling design

We carried out ant sampling during Summer 2013 (January and February). In each forest-grassland ecotone we established one parcel (10 x 70 m) inside each habitat (i.e. predominantly forest and predominantly grassland). Each parcel was 35 m apart from the edge between habitats, and about 70 m from each other. In each parcel we placed fourteen baited sample points (seven with honey and seven with tuna fish in oil) over paper cards (10 x 10 cm), 10 m from each other (two rows of seven baits), left to attract ants for 1 hour (Fig 1B). After this, ants on each bait were stored in plastic bags with ethyl acetate and then preserved in 80% ethanol. All ant individuals were taken to Laboratório de Ecologia de Interações (LEIN) in Universidade Federal do Rio Grande do Sul (UFRGS) for further processing.

Ant genera identification was based on dichotomous keys [43]. Specific literature was used for species classification, and direct comparisons were done with specimens in scientific ant collections in LEIN and the Entomological Collection Padre Jesus Santiago Moure of the Universidade Federal do Paraná (DZUP). Morphospecies determination followed the standard practice of LEIN, where vouchers are deposited.

### Environmental and spatial variables

At the local scale, we recorded soil surface air temperature (°C) and air moisture (%) at the moment of ant sampling in both forests and grasslands. We used two data loggers (HOBO Pro V2 Temp/RH Onset) per parcel recording data at each 5 min for one hour. Habitat structure variables were collected at each habitat independently, according to their physiognomy. In forest parcels, we evaluated leaf-litter depth (cm) and canopy openness (%) at three equidistant points 30 m from each other (one point at 5 m, one at 35 m and another at 65 m on parcel). In grassland parcels we measured herbaceous vegetation height (cm) and shrub and tree density (amount of branches and leaves touching a 1.5 m height pole at 10 cm radius) also at three equidistant points. All local predictor variables at each parcel (forest and grassland) were averaged among the points, and are available in S1 and S2 Tables, respectively.

At the regional scale, we considered two categories of environmental variables: climate and geomorphometry. Data were extracted for the nine sampling sites. Climate variables (annual mean temperature, temperature seasonality, minimum temperature of coldest month, annual precipitation and precipitation seasonality) were obtained from WorldClim—Global Climate Data (http://www.worldclim.org) [44]. Temperature and precipitation seasonality summarizes the monthly variation during the year. Mean altitude of each site was used as a geomorphometry variable, obtained from Shuttle Radar Topographic Mission data available from the CIAT-CSI database [45]. Descriptions of all sites in terms of their regional predictor variables are available in S3 Table.

Some predictor variables might be highly correlated and influence the analysis. We used the Variance Inflation Factor (VIF) to detect multicollinearity among environmental variables [46]. We calculated VIF for all predictors, for both scales, and selected only those with VIF ≤ 3, which indicate insignificant multicollinearity [47,48]. Thus, for local scale analysis, air moisture was removed (S4 Table), and for regional scale analysis, minimum temperature of the coldest month and mean altitude were removed (S5 Table). VIF values were calculated with the *fmsb* package in *R* [49].

Finally, the spatial matrix was arranged from geographical coordinates (latitude and longitude) of one central point between ecotones on each site (S6 Table). This matrix was transformed into spatial data by the Principal Coordinates of Neighbor Matrices method (PCNM) [50]. Five PCNM vectors were generated using the *pcnm* function of the *vegan* package [51].

### Data analysis

For the local scale analysis, we searched for predictors of ant communities in forests and grasslands separately. Thus, we had 17 sampling units of forest and 17 of grassland. At the regional scale, we considered the site (data from two ecotones) as a sampling unit, totaling nine samples. All analyses presented here were implemented in the *R* software environment [49].

#### Ant phylogenetic tree

Currently, there is no complete species-level ant phylogeny available. We considered phylogenetic relationships among ant genera from the phylogeny of Moreau & Bell [52] and complemented this database with the phylogenetic relationships within Myrmicinae proposed by Ward et al. [53]. These two publications with time-calibrated phylogeny were used to build a phylogenetic tree for the ant communities found in the present study. We built this tree at genus-level in the software Phylocom 4.2 [54]. Then, all species were inserted in this tree as polytomies. After that, we randomly generated 1000 potential trees considering the relationships among species within each genus as phylogenetic uncertainty in the software Sunplin [55] (see an example of one of the 1000 phylogenetic trees in S1 Appendix).

#### Taxonomic and phylogenetic diversity

We characterized the taxonomic diversity for each forest and grassland (local scale), and each site (regional scale) using species richness (the number of species in each habitat or site) and species diversity (Simpson index), henceforth *S* and *D*, respectively. Phylogenetic diversity was calculated with Faith’s phylogenetic diversity (*PD*) and Rao’s quadratic entropy (*PR*, which is equivalent to the Simpson index). *PD* was the sum of branch lengths of the phylogenetic tree linking all the species represented in each community [56] and *PR* was calculated considering phylogenetic distance among species in each habitat or site weighted on the proportion of the occurrences of ant species [57]. We used these two metrics because *PD* is not an abundance (or occurrence) weighted index, while *PR* is (as is *D*). We calculated *PD* and *PR* for the 1000 phylogenetic trees generated by randomization (as described above) and used the mean value of these metrics for each habitat or site in further analyses. *PD* was calculated with the *picante* package [58] and *D* and *PR* with *SYNCSA* [59].

#### Taxonomic and phylogenetic composition

Principal Coordinates Analysis (PCoA) based on the Bray-Curtis index among sampling units was used for the ordination of species taxonomic composition in forests and grasslands (local scale) and sites (regional scale). We used the *Adonis* function (permutation-based multivariate analysis of variance) with 9,999 permutations to examine differences between habitats (local scale) and physiographic regions (regional scale).

To explore ant phylogenetic composition between habitats (local scale) and physiographic regions (regional scale), we performed an analysis of Principal Coordinates of Phylogenetic Structure (*PCPS*) [60]. *PCPS* analysis represents the variation in phylogenetic composition across environmental gradients with eigenvectors (ordination vectors–*PCPS*). This method has already been applied to different taxonomic groups with relatively well-established phylogenetic relationships among species, such as birds [61], amphibians [62] and plants [63–65], and it is briefly described below.

First, the matrix with phylogenetic patristic distance between species (matrix **D**) is transformed into a matrix with pairwise phylogenetic similarities between species (matrix **S**). Then, the phylogenetic weights of taxa are calculated by fuzzy weighting [66] through standardization by the marginal totals within the columns of matrix **S**, generating the matrix **Q**. The matrix **Q** considers the phylogenetic relationships among the taxa, reflecting the evolutionary history shared between one taxon compared with all others [67]. Then, the matrix **Q** is finally multiplied by the matrix of species occurrences by communities (matrix **W**) to generate the matrix of phylogeny-weighted species composition (matrix **P**). *PCPS* vectors are extracted through Principal Coordinates Analysis (*PCoA*) based on matrix **P**, resulting in eigenvectors that describe the variation of phylobetadiversity across environmental gradients. *PCPS* is able to capture phylobetadiversity patterns from both basal and more terminal nodes associated with specific communities [67]. Ultimately, the phylobetadiversity pattern found is tested against null models (*taxa shuffle*) based on permutations of phylogenetic relationships among species (9,999 permutations) while species composition is kept the same across the communities. Thus, a significant probability value of *taxa shuffle* means that the association between species distribution and environmental gradients is structured by the phylogenetic relationships among species. More details about these procedures can be accessed in [67].

In our study, we performed *PCPS* analysis for each of the 1000 ant phylogenetic trees generated by randomization (previously described). We then presented the proportion of significant/non-significant *PCPS* (i.e. n-trees out of 1000 trees that returned *p* ≤ 0.05) and discussed the results. This is the first time that the analysis of Principal Coordinates of Phylogenetic Structure has been applied to explore phylobetadiversity of data while treating the relationships among species within each genus as phylogenetic uncertainty. We used the *vegan* package for PCoA and *Adonis* function, and the *PCPS* package for PCPS analysis [68].

#### Local predictors

First we tested whether metrics of taxonomic and phylogenetic diversity differ between habitat types (17 units of forests and 17 units of grasslands) through generalized linear mixed models (GLMMs). At this scale, habitat type was used as a fixed factor and site (nine units) was used as a random factor (y ~ habitat type + (1|site)). We assumed a Poisson distribution for *S* and Gaussian distributions for *D*, *PD* and *PR* metrics fitted with the *fitdist* function in the *fitdistrplus* package (based on maximum likelihood estimation) [69]. We applied ANOVA to test the significance and obtained the χ^2^ and *p*-values for each model.

We also fitted GLMMs with the same data distribution to test the responses of taxonomic and phylogenetic metrics to the local environmental variables in each habitat separately. We selected the model(s) that best explained the patterns based on the Akaike’s information criterion corrected for small samples (AICc) [70]. For each response variable, we applied the complete additive model (with all variables), simple models with interaction (only between two variables), and the null models (y ~ 1 + (1|site)). The models with ΔAICc ≤ 2 were considered viable to explain the observed patterns. Additionally we calculated the conditional coefficient of determination R^2^_(c)_ for the selected models. The conditional R^2^ represents the variance explained by both fixed and random factors [71]. All selected models were submitted to residual analysis to evaluate the adequacy of the error distribution. GLMMs were performed using the *glmer* function for *S* and *lmer* function for *D*, *PD* and *PR*, both with the *lme4* package [72]. The model selections based on AICc criteria and the conditional R^2^ were implemented with the *MuMIn* package.

To verify whether environmental variables might be influencing species composition, we performed a forward selection analysis based on redundancy analysis (RDA) for forests and grasslands, separately. To reduce the effect of rare species, singletons (i.e. species with only one occurrence) were removed from this analysis [73]. Variables with *p* ≤ 0.05 were selected as significant to explain the variation in ant composition. Forward selection was performed with the *vegan* package.

#### Regional predictors

At the regional scale, we used the mean value of the metrics between each forest-grassland ecotone for each site, totaling nine values. We did this since one site from the CC region had only one ecotone studied. In addition, each regional variable was obtained on a site level and not on an ecotone level (data from WorldClim). Then, we first tested whether metrics of taxonomic and phylogenetic diversity differ among physiographic regions using GLMMs as previously above. In these models, we used the region as a fixed factor while the sites were entered as a random factor (y ~ region + (1|site)). We apply the same distribution errors for each metric from the local scale and obtained the χ^2^ and *p*-values. When a model showed significant differences, we performed Tukey post-hoc tests for comparisons among means with the *multcomp* package [74].

Subsequently, we evaluated the responses of taxonomic and phylogenetic metrics to regional environmental variables. To select the most suitable models we applied Akaike’s information criterion corrected for small samples (AICc) as previously described for the local scale, considering the complete additive model, simple models with interaction, and the null model for each metric separately.

For ant taxonomic composition, we applied a forward selection analysis based on RDA following exactly the same procedures as explained for the local scale. Further, we verified whether, besides environmental variables, spatial variables also influenced species composition at the regional scale through partitioning analysis. For this, we submitted the PCNMs matrix also to forward selection. Then, we performed a variation partitioning analysis dividing the contribution of the total variance of species composition into four fractions, and tested their significance: [a] the component only explained by the environment (independent of the spatial variation); [b] the component explained by the environment that is also spatially structured (spatially structured environmental filtering); [c] the component explained only by space (independent of the environmental variation); and [d] the residual variation [75]. The variation partitioning analysis was carried out with the *varpart* function in the *vegan* package.

## Results

### Ant fauna

We sampled 10,906 ants, belonging to six subfamilies, 23 genera and 85 morphospecies (S2 Appendix). The richest subfamilies were Myrmicinae (46 spp.) and Formicinae (19 spp.), and the richest genera were *Pheidole* (18 spp.), *Camponotus* and *Crematogaster* (nine spp. each) and *Brachymyrmex* (eight spp.). *Pheidole radoszkowskii* Mayr, 1884, *Pheidole* nr. *pubiventris* Mayr, 1887 and *Solenopsis invicta* Buren, 1972 were the most frequent species (66, 65 and 35 occurrences, respectively). Sixty species were sampled in forests and 63 in grasslands. Twenty-two species were exclusive of forests while 25 were exclusive of grasslands. The SS region had the most ant species (53 spp.), followed by CA (48 spp.) and CC (40 spp.). Overall, 27 singletons were collected.

### Taxonomic and phylogenetic diversity

**Fig 2:**
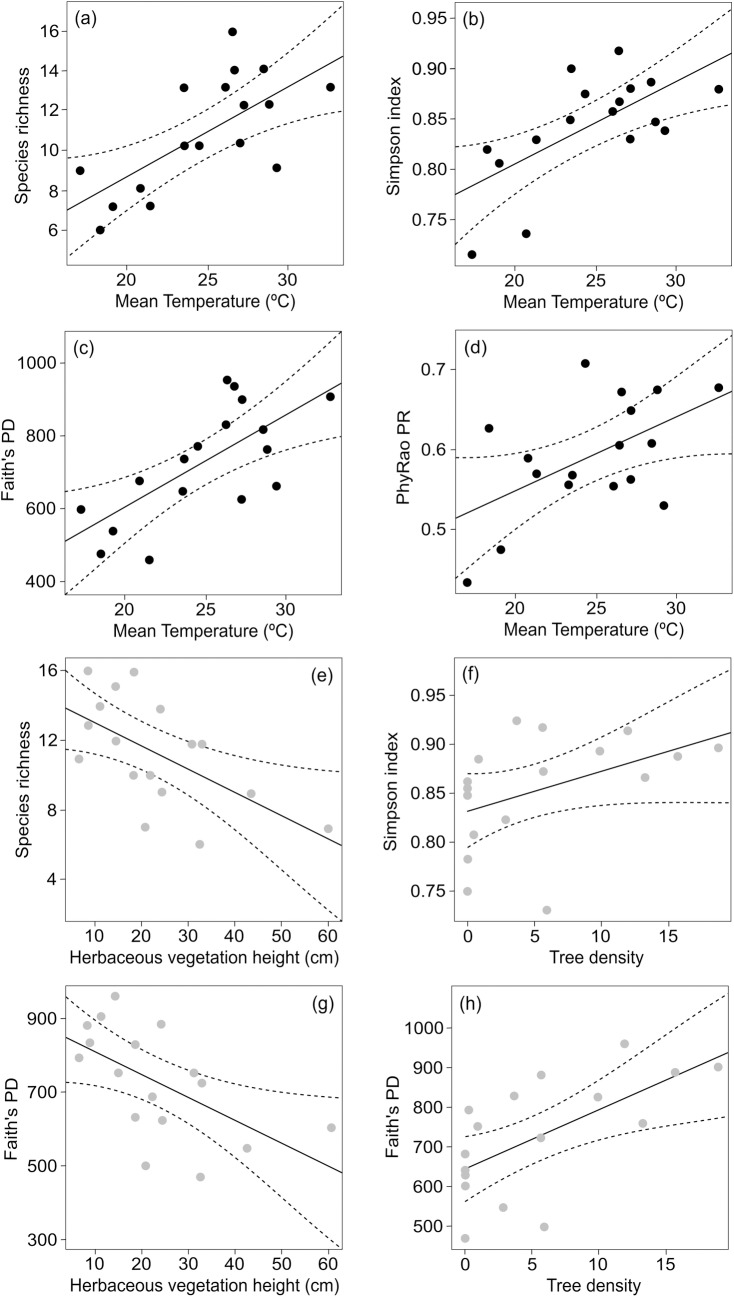
Relationship between local variables and ant taxonomic and phylogenetic metrics in forest-grassland ecotones in South Brazil. The best generalized linear mixed models (ΔAICc = 0.0) using sites as a random factor: (a) to (d) plots represent relationships in forests (black dots) and (e) to (h) plots represent relationships in grasslands (grey dots).

Taxonomic and phylogenetic metrics did not differ between forests and grasslands (*S*: χ^2^ = 0.27, *p* = 0.61; *D*: χ^2^ = 0.38, *p* = 0.54; *PD*: χ^2^ = 0.009, *p* = 0.93; *PR*: χ^2^ = 0.09, *p* = 0.76). However, we found specific local variables explaining these metrics at each habitat. In forests, we found a positive relationship between soil surface air temperature and *S* (R^2^_(c)_ = 0.27, Fig 2A), *D* (R^2^_(c)_ = 0.49, Fig 2B), *PD* (R^2^_(c)_ = 0.51, Fig 2C) and *PR* (R^2^_(c)_ = 0.67, Fig 2D), meaning that sites with higher temperatures in the sampling moment also presented higher taxonomic and phylogenetic diversity (Table 1).

**Table 1:** Best-supported models (GLMMs) in forests.

Distribution	Response variable	Model	AICc	ΔAICc	df	Weight	R^2^_(c)_
Poisson	Species richness (*S*)						
		MTF	86.1	0.0	3	0.455	0.27
Gaussian	Simpson index (*D*)						
		MTF	-50.9	0.0	4	0.619	0.49
Gaussian	Faith's PD (*PD*)						
		MTF	218.8	0.0	4	0.729	0.51
Gaussian	PhyRao (*PR*)						
		MTF	-38.6	0.0	4	0.420	0.67

Best-supported models with ΔAICc ≤ 2 retained in forest habitats from forest-grassland ecotones in South Brazil. MTF-Soil Surface Air Mean Temperature of Forests (°C).

In grasslands, we found *S* (R^2^_(c)_ = 0.27, Fig 2E), *D* (R^2^_(c)_ = 0.31) and *PR* (R^2^_(c)_ = 0.14) negatively related to herbaceous vegetation height, and *S* (R^2^_(c)_ = 0.20), *D* (R^2^_(c)_ = 0.75, Fig 2F) and *PD* (R^2^_(c)_ = 0.82) positively associated with tree density (Table 2). Furthermore, we also found, as possible models, herbaceous vegetation height combined with tree density explaining *S* (R^2^_(c)_ = 0.34), *D* (R^2^_(c)_ = 0.64) and *PD* (R^2^_(c)_ = 0.85, Fig 2G and 2H). That is, grasslands with taller herbaceous vegetation presented lower numbers of ant species and phylogenetic diversity while grasslands with higher tree density increased ant taxonomic and phylogenetic diversity. *PR* was the only metric where the null model was selected (Table 2).

**Table 2:** Best-supported models (GLMMs) in grasslands.

Distribution	Response variable	Model	AICc	ΔAICc	df	Weight	R^2^_(c)_
Poisson	Species richness (*S*)						
		HVE	89.1	0.0	3	0.268	0.27
		TRD	90.0	0.9	3	0.173	0.20
		HVE + TRD	90.3	1.1	4	0.154	0.34
Gaussian	Simpson index (*D*)						
		TRD	-45.3	0.0	4	0.278	0.75
		HVE	-44.7	0.6	4	0.202	0.31
		HVE + TRD	-43.9	1.5	5	0.133	0.64
Gaussian	Faith's PD (*PD*)						
		HVE + TRD	215.1	0.0	5	0.418	0.85
		TRD	215.8	0.7	4	0.288	0.82
Gaussian	PhyRao (*PR*)						
		Null Model	-49.4	0.0	3	0.336	Null
		HVE	-48.3	1.1	4	0.197	0.14

Best-supported models with ΔAICc ≤ 2 retained in grassland habitats from forest-grassland ecotones in South Brazil. HVE-Herbaceous Vegetation Height (cm); TRD-Tree Density.

**Fig 3:**
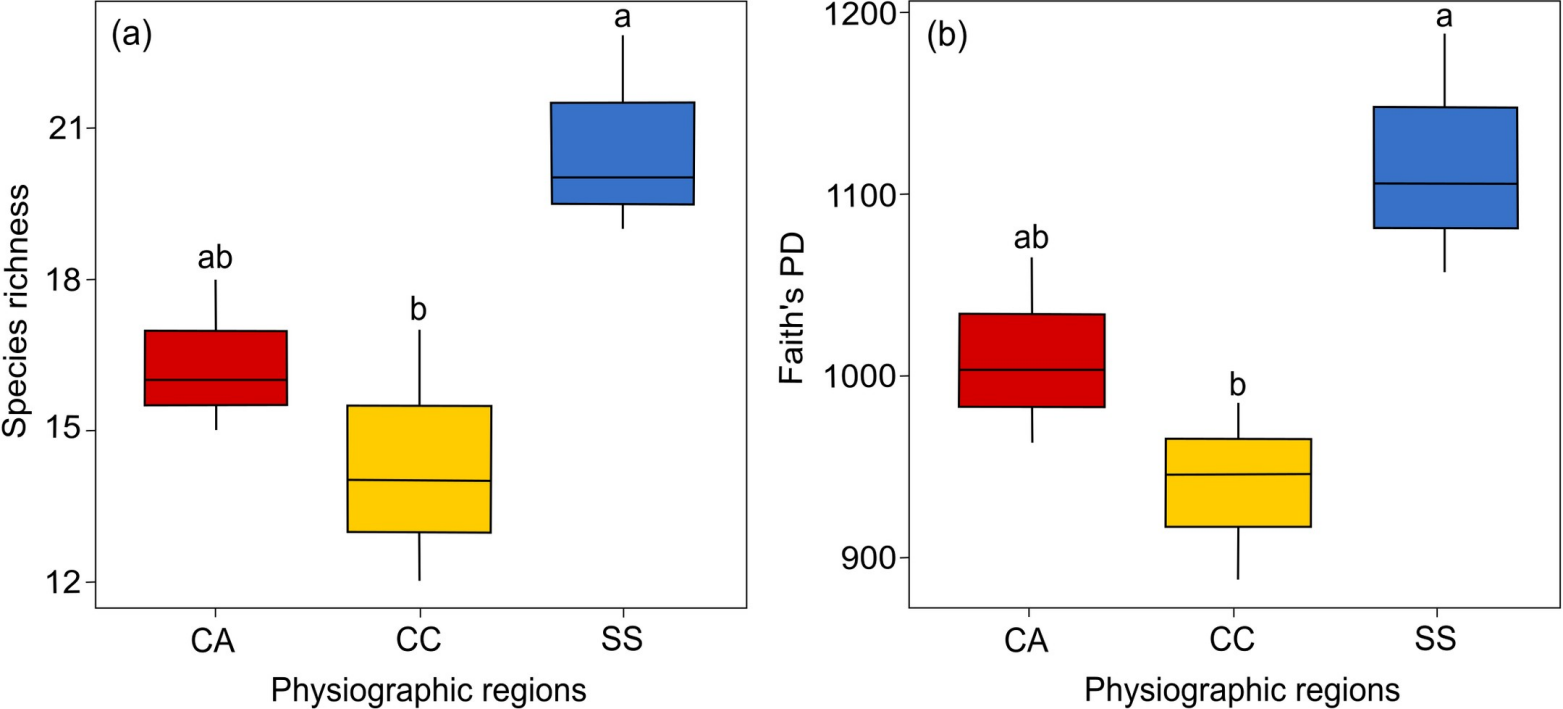
Boxplot showing ant species richness and phylogenetic diversity among physiographic regions in South Brazil. Forest-grassland ecotones from the SS region had more (a) ant species and (b) phylogenetic diversity than the CC region. Different regions of Rio Grande do Sul state: Campanha region—CA (red boxes), Campos de Cima da Serra region—CC (yellow boxes) and Serra do Sudeste region—SS (blue boxes). Tukey post-hoc tests (a) CA:CC (*p* = 0.50); CA:SS (*p* = 0.11); CC:SS (*p* = 0.02) and (b) CA:CC (*p* = 0.34); CA:SS (*p* = 0.13); CC:SS (*p* = 0.01).

At the regional scale, we found significant differences among regions for *S* (χ^2^ = 15.6, *p* = 0.05, Fig 3A) and *PD* (χ^2^ = 605.4, *p* < 0.001, Fig 3B), with ecotones from the SS region exhibiting more ant species and phylogenetic diversity than the CC region. At this scale, we did not find suitable models using our regional environmental variables to explain ant diversity. Only null models met the model selection criteria (Table 3).

**Table 3:** Best-supported models (GLMMs) at regional scale.

Distribution	Response variable	Model	AICc	ΔAICc	df	Weight	R^2^_(c)_
Poisson	Species richness (*S*)						
		Null Model	53.2	0.0	2	0.527	Null
Gaussian	Simpson index (*D*)						
		Null Model	-35.9	0.0	3	0.775	Null
Gaussian	Faith's PD (*PD*)						
		Null Model	113.9	0.0	3	0.846	Null
Gaussian	PhyRao (*PR*)						
		Null Model	-23.3	0.0	3	0.825	Null

Best-supported models with ΔAICc ≤ 2 retained in forest-grassland ecotones at the regional scale in South Brazil.

### Taxonomic and phylogenetic composition

**Fig 4:**
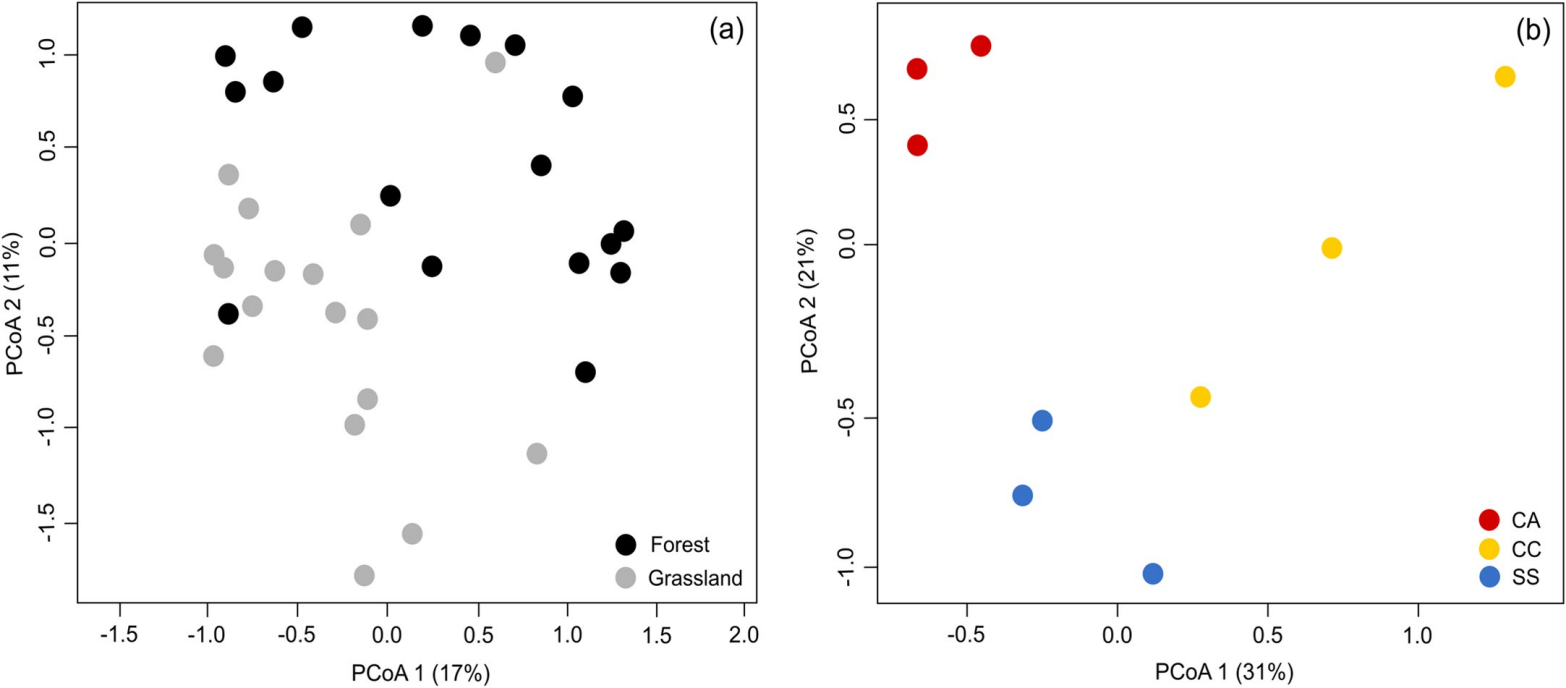
Ordination diagrams of ant species composition. (a) Principal Coordinates Analysis (PCoA) of forest-grassland ecotones based on ant species composition (frequency matrix) with Bray-Curtis similarity index. Black dots represent forest sampling sites and grey dots grasslands sampling sites. (b) PCoA at the regional scale with nine sites belonging to three physiographic regions. Different regions of Rio Grande do Sul state: Campanha region—CA (red dots), Campos de Cima da Serra region—CC (yellow dots) and Serra do Sudeste region—SS (blue dots).

Ant taxonomic composition was clearly different between forests and grasslands with 28% of the variation explained by the first two PCoA axes (Adonis: F = 2.67, *p* < 0.01, Fig 4A). However, we did not find differences in ant phylogenetic composition based on all 1000 phylogenetic trees permuted in PCPS analysis (*p*_*(taxa shuffle*)_ > 0.05 for all 1000 phylogenetic trees). The forward selection did not retain any local environmental variables associated with species composition either in forests or grasslands, only the habitat variable.

At the regional scale, we found differences in ant taxonomic composition among different physiographic regions (52% of the variation explained by the first two PCoA axes; Adonis: F = 2.35, *p* < 0.01, Fig 4B), but we also did not find differences in phylogenetic composition (*p*_(*taxa shuffle*)_ > 0.05 for all 1000 phylogenetic trees). The forward selection retained only annual mean temperature affecting ant taxonomic composition (df = 1, AIC = 54.838, F = 1.84, *p* = 0.02).

After submitting all five PCNM vectors to forward selection, only two were retained: PCNM 2 (df = 1, AIC = 54.803, F = 1.87, *p* = 0.01) and PCNM 1 (df = 1, AIC = 54.283, F = 1.94, *p* = 0.02). Thus, variation partitioning analysis was carried out with only one environmental and two spatial variables. Overall, environmental and spatial variables explained 27% of the variation in ant taxonomic composition, i.e. 73% was unexplained (residuals). Of the 27% explained, 6% was purely environmental, 18% purely spatial and 3% spatially structured environmental variation. However, the purely environmental proportion was not significant, i.e. the variation in ant composition found among physiographic regions is largely due to spatial effects (Table 4).

**Table 4:** Variation partitioning analysis.

Fractions of variation	R^2^	R^2^_ajd_	F	*p*
[a+b] Environmental + shared	0.21	0.09	1.84	0.02
[b+c] Spatial + shared	0.40	0.20	2.03	0.001
[a+b+c]	0.54	0.27	1.98	0.001
[a] Only environmental		0.06	1.53	0.12
[b] Environment spatially structured		0.03		
[c] Only spatial		0.18	1.84	0.01
[d] Residual		0.73		

Variation partitioning showing the relative influence of environmental variables [a] (only annual mean temperature), spatial variables [c] (PCNM 1 and 2), spatially structured environmental [b] and residual variation [d] on ant taxonomic composition in forest-grassland ecotones in South Brazil.

## Discussion

At a local scale, our study did not show differences in ant diversity between adjacent grasslands and forests, corroborating both Pinheiro et al. [31] and Klunk et al. [32] for South Brazil, even when more than one stratum (ground, leaf litter and arboreal) is considered (as discussed by Klunk et al. [32]). Overall, studies have showed open ecosystems such as grasslands/savannas harboring higher ant diversity than forests in ecotones or landscape mosaics, for many regions of the world (e.g. [76–78]). In Brazilian neotropical savannas this pattern also seems to occur, as showed by Camacho & Vasconcelos [79]. Mirroring the distinct forest and grassland plant communities, we found distinct ant communities in these habitats [76–78]. Despite short distances between sampling sites in different habitats (about 70 m), environmental filtering probably sort those species more adapted to or with a higher advantage when inhabiting each specific habitat [24]. We did not detect differences in ant phylogenetic composition between forest and grasslands, suggesting that no specific lineages evolved or have adapted to each environment in this region along its evolutionary history.

Within forests, we found the ground surface temperature as a driver of the local foraging ant diversity (both taxonomic and phylogenetic). This means that at higher temperatures during the day, or on hotter days, more forest ant species and ant lineages are actively exploring the environment. Ants are poikilothermic organisms, so their temperature depends on the surrounding environment, which determines their metabolic rates and foraging speed [80]. Closed-canopy habitats, such as forests, are typically shaded and cooler than open-canopy ecosystems (in our study, grasslands: mean 29.9°C with max 36.2°C; forests: mean 24.8°C with max 32.7°C), in addition to offering buffered microclimate conditions to the biota [81]. Thus, forests and grasslands may present ant species with different thermal niches [82], with forest ant communities more sensitive to the daily thermal oscillation in comparison to grassland species. Further studies should clarify this topic in detail with experiments and field observations.

In grasslands, ant diversity was driven by habitat structural properties. The diversity of ant species and evolutionary histories decreased with the height of herbaceous vegetation. Tall grassland vegetation usually characterizes ecosystems with low levels of disturbances (e.g. grazing and fire), where the biomass of a few dominant plant species, standing dead mass and a dense litter layer accumulates [27,83]. In this system, plant functional groups such as forbs (i.e. plants that typically present attractive resources to fauna) may be outcompeted by the dominant tussock grasses and disappear below them, decreasing the total local plant diversity. This process gradually modifies natural habitat characteristics and could decrease ant diversity due to habitat simplification and reduction in resource diversity [84]. In Neotropical Brazilian savannas (Cerrado biome), where fire helps to maintain biodiversity, fire suppression results in severe reduction of both plant and ant species (27% and 35% respectively [35]).

Another possible explanation for the negative relation between ant diversity and the height of herbaceous vegetation is the alteration of ant competitive interactions at the community level according to the grassland disturbance levels [85]. A relief in disturbance intensity (i.e. leading to taller vegetation) might trigger negative competitive interactions among ants, decreasing species coexistence. On the contrary, moderate or intense grazing (i.e. leading to shorter vegetation) might allow greater ant diversity by diminishing the dominance of particular species, as discussed by Moranz et al. [86] for tallgrass prairies from central North America. Furthermore, if we assume ant phylogenetic diversity as a potential proxy for ant functional diversity [20,29,30], our results are likely to indicate a decline of the ecological functions performed by ants in tall homogeneous grasslands.

Interestingly, we also found tree density on grasslands promoting ant species and phylogenetic diversity. The establishment of trees over the grass matrix progressing from the forest/grassland edge represents a classical forest expansion process [87]. Such process can clearly amplify habitat environmental/spatial heterogeneity, and thus the availability of ecological niches and resources for ants, affecting the dominance hierarchy [36]. By locally changing grassland environmental conditions at the ground level (e.g. solar incidence), it is plausible to expect treelet density allows species with different requirements to coexist [88], i.e. both forest and grassland-prone species. Similarly to our considerations on the correlations between vegetation height and grassland management, forest expansion processes usually take place on non-grazed or slightly grazed grasslands [87]. Knowledge about the responses of South Brazilian ant communities to grassland management and their relation with specific plant structures is strongly limited [37] and an intensive research effort on this topic is needed.

At a regional scale, we detected distinct ant species composition among ecotones in the three different physiographic regions in South Brazil (CA, CC and SS), which was mostly structured by space. Similarly, Arnan et al. [20] found spatial effects assembling ant communities across western and central Europe. Differences in ant species composition in South Brazilian grasslands were already reported for the CC region compared to the SS and CA regions by Dröse et al. [37]. However, we did not detect patterns in ant phylogenetic composition among the physiographic regions, indicating no divergence in specific ant lineages at this scale. Taxonomic differences, but not phylogenetics, indicate that macroscale variations in ant communities in South Brazil are primarily at the species rather than genus or subfamily level. Ant species can be strongly affected by spatial variables because of the low mobility and dispersal capacity of gynes [19]. Also, besides this limitation, different historical processes and landscape features may contribute to community dissimilarity [89]. In our study, higher altitudes and formation of gorges (CC region) and valleys (SS region) may have acted as dispersal barriers, increasing ant species dissimilarity among regions, but not affecting widespread ant lineages. Ultimately, although many studies report the water-energy dynamic hypothesis as elucidating ant macroscale patterns [12,13,18,38,39], the environmental variables considered in this study did not explain our regional patterns. This can be attributed to (i) the omission of important spatially structured environmental variables (e.g. landscape habitat loss [84]), or even (ii) low site replication at the regional scale (n = 9) that could be increasing the probability of committing Type II errors. Furthermore, stochastic processes might be at play in structuring these ant communities, meaning that species with similar ecological traits are allocated to the physiographic regions mostly by ecological drift and dispersal [19,20].

The standardized baiting sampling employed in this study provided fast and low cost surveys of ant communities from 34 forests and grasslands throughout a geographic extent of more than 553 km traveled in less than two months in the southernmost part of Brazil. This rapid ant assessment presented sufficient resolution to detect taxonomic and phylogenetic patterns in forest-grassland ecotones across different regions. Nevertheless, we cannot rule out completely that baiting may be leading to a biased assessment of communities in cases when behaviorally dominant ant species impede lonely or subordinate species from reaching the bait [90,91]. In this context, cryptic (confined to litter and soil) and rare ant species and lineages associated with forest or grassland habitats could have been underestimated in this study, hiding some patterns (e.g. phylogenetic composition). However, we do not have sufficient field evidence and knowledge about the species pool in this region to suggest this to be at work. In the Cerrado biome, pitfall traps and winkler extractions collected more ant species than sardine baits in forest and savanna physiognomies [92]. Despite that, baiting was as efficient as the other sampling methods to detect differences in species composition between physiognomies, indicating it to be adequate for studies comparing distinct habitats or conditions.

## Conclusions

Our study unveils important aspects of ant community assembly and drivers in natural forest-grassland ecotones in South Brazil, considering taxonomic and phylogenetic perspectives, and may serve as a reference to other studies in these ecological transition systems worldwide. Here we showed that forests and grasslands are similar regarding ant diversity at ground level, but considerably different in terms of species composition (but not phylogenetic). In forests, the soil surface air temperature predicts foraging ant diversity. In grasslands, the height of herbaceous vegetation reduces ant diversity while treelet density from forest expansion processes clearly increases it. At a regional scale, space explained the most of the variance in species composition, and no environmental variables sufficiently explained ant diversity patterns at this scale. These results call for attention to the importance of these natural habitats and their biodiversity. All different habitat physiognomies from different regions of southern Brazil should warrant equally distributed conservation efforts to maximize biodiversity, but special care should be devoted to grasslands that are currently under major threat of conversion to other land use types.

## Supporting information

S1 Appendix Phylogenetic tree from the 85 ant species collected in forest-grassland ecotones in South Brazil. An example of one of the 1000 phylogenetic trees built in the software Sunplin considering the relationships among species as phylogenetic uncertainly. Scale bar in millions of years before the present.
(PDF)

S2 Appendix List of ant species recorded in forest-grassland ecotones in South Brazil. Numbers represent the total number of occurrences in the three physiographic regions (CA-Campanha; CC-Campos de Cima da Serra; SS-Serra do Sudeste) and habitats (F-forest; G-grassland). *New record to Rio Grande do Sul state, Brazil.
(PDF)

S1 Table Local environmental variables sampled in forests from forest-grassland ecotones in Rio Grande do Sul state, Brazil. COP-Canopy Openness (%); LIT-Litter Depth (cm); MOF-Air Moisture of Forests (%); MTF-Soil Surface Air Mean Temperature of Forests (°C).
(PDF)

S2 Table Local environmental variables sampled in grasslands from forest-grassland ecotones in Rio Grande do Sul state, Brazil. HVE-Herbaceous Vegetation Height (cm); MOG-Air Moisture of Grasslands (%); MTG-Soil Surface Air Mean Temperature of Grasslands (°C); SHD-Shrub Density; TRD-Tree Density.
(PDF)

S3 Table Regional variables obtained from WorldClim (V1 to V5) and Shuttle Radar Topographic Mission (V6) to three different physiographic regions from Rio Grande do Sul state, Brazil. V1-Annual Mean Temperature (°C); V2-Temperature Seasonality (°C); V3-Minimum Temperature of Coldest Month (°C); V4-Annual Precipitation (mm); V5-Precipitation Seasonality (%); V6-Mean Altitude (m).
(PDF)

S4 Table **Variance inflation factor (VIF) table with local environmental variables sampled in (a) forests and (b) grasslands from forest-grassland ecotones in Rio Grande do Sul state, Brazil. Bold numbers means multicollinearity between variables (VIF > 3).** COP-Canopy Openness (%); LIT-Litter Depth (cm); MOF-Air Moisture of Forests (%); MTF-Soil Surface Air Mean Temperature of Forests (°C); HVE-Herbaceous Vegetation Height (cm); MOG-Air Moisture of Grasslands (%); MTG-Soil Surface Air Mean Temperature of Grasslands (°C); SHD-Shrub Density; TRD-Tree Density.
(PDF)

S5 Table Variance inflation factor (VIF) table with regional variables obtained to three different physiographic regions from Rio Grande do Sul state, Brazil. **Bold numbers means multicollinearity between variables (VIF > 3).** V1-Annual Mean Temperature (°C); V2-Temperature Seasonality (°C); V3-Minimum Temperature of Coldest Month (°C); V4-Annual Precipitation (mm); V5-Precipitation Seasonality (%); V6-Mean Altitude (m).
(PDF)

S6 Table Geographical coordinates obtained from one central point between ecotones in each site. (PDF)
