# Supplementary material for: Local and regional drivers of ant communities in forest-grassland ecotones in South Brazil: A taxonomic and phylogenetic approach
Source: PLoS One. 2019 Apr 11;14(4):e0215310. doi: 10.1371/journal.pone.0215310 (PMC6459495; doi:10.1371/journal.pone.0215310)

S1 Appendix. Phylogenetic tree from the 85 ant species collected in forest-grassland ecotones in South Brazil.

An example of one of the 1000 phylogenetic trees built in the software Sunplin considering the relationships among species as phylogenetic uncertainly. Scale bar in millions of years before the present.

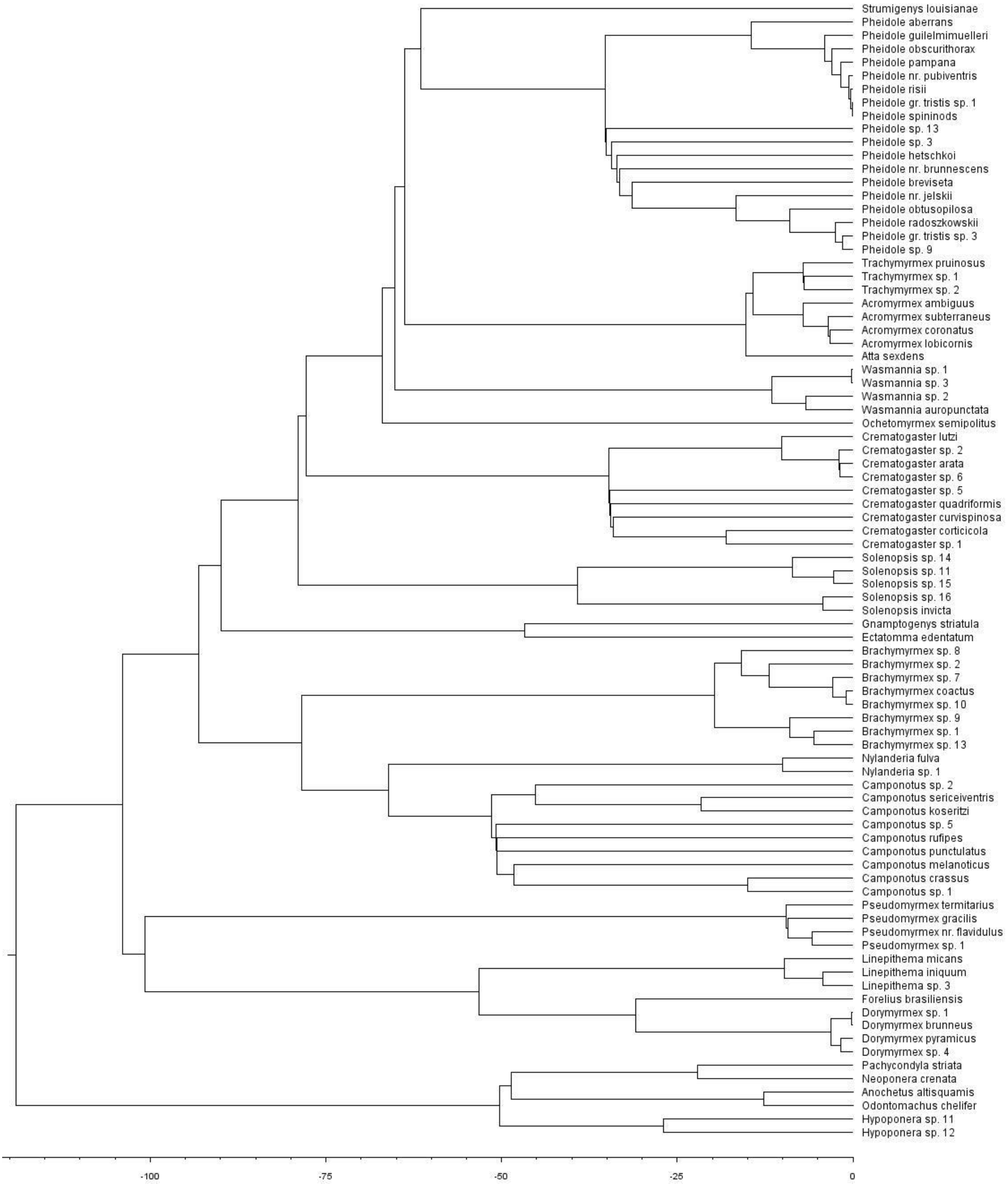

Supplement: S1 Appendix — An example of one of the 1000 phylogenetic trees built in the software Sunplin considering the relationships among species as phylogenetic uncertainly. Scale bar in millions of years before the present. (PDF) [file pone.0215310.s001.pdf]
