# Supplementary material for: Local and regional drivers of ant communities in forest-grassland ecotones in South Brazil: A taxonomic and phylogenetic approach
Source: PLoS One. 2019 Apr 11;14(4):e0215310. doi: 10.1371/journal.pone.0215310 (PMC6459495; doi:10.1371/journal.pone.0215310)
Supplement: S2 Appendix — Numbers represent the total number of occurrences in the three physiographic regions (CA-Campanha; CC-Campos de Cima da Serra; SS-Serra do Sudeste) and habitats (F-forest; G-grassland). *New record to Rio Grande do Sul state, Brazil. (PDF) [file pone.0215310.s002.pdf]

**S2 Appendix. List of ant species recorded in forest-grassland ecotones in South Brazil.** Numbers represent the total number of occurrences in the three physiographic regions (CA-Campanha; CC-Campos de Cima da Serra; SS-Serra do Sudeste) and habitats (F-forest; G-grassland). *New record to Rio Grande do Sul state, Brazil.

| **Ant species composition** | **CA** | | **CC** | | **SS** | |
|---|---|---|---|---|---|---|
| | **F** | **G** | **F** | **G** | **F** | **G** |
| **Dolichoderinae** | | | | | | |
| *Dorymyrmex brunneus* Forel, 1908 | 0 | 5 | 0 | 0 | 0 | 0 |
| *Dorymyrmex pyramicus* (Roger, 1863) | 0 | 1 | 0 | 0 | 0 | 0 |
| *Dorymyrmex* sp. 1 | 0 | 0 | 0 | 0 | 0 | 1 |
| *Dorymyrmex* sp. 4 | 0 | 0 | 0 | 0 | 0 | 2 |
| *Forelius brasiliensis* (Forel, 1908) | 0 | 0 | 0 | 0 | 0 | 1 |
| *Linepithema iniquum* (Mayr, 1870) | 0 | 0 | 1 | 0 | 15 | 7 |
| *Linepithema micans* (Forel, 1908) | 1 | 7 | 5 | 10 | 0 | 1 |
| *Linepithema* sp. 3 | 0 | 0 | 0 | 0 | 0 | 2 |
| **Ectatomminae** | | | | | | |
| *Ectatomma edentatum* Roger, 1863 | 1 | 7 | 0 | 0 | 1 | 0 |
| *Gnamptogenys striatula* Mayr, 1884 | 7 | 0 | 1 | 0 | 6 | 3 |
| **Formicinae** | | | | | | |
| *Brachymyrmex coactus* Mayr, 1887 | 0 | 0 | 0 | 0 | 0 | 3 |
| *Brachymyrmex* sp. 1 | 1 | 4 | 0 | 0 | 1 | 17 |
| *Brachymyrmex* sp. 2 | 0 | 0 | 0 | 0 | 1 | 4 |
| *Brachymyrmex* sp. 7 | 0 | 0 | 1 | 9 | 0 | 0 |
| *Brachymyrmex* sp. 8 | 1 | 0 | 5 | 0 | 5 | 0 |
| *Brachymyrmex* sp. 9 | 0 | 0 | 1 | 0 | 0 | 0 |
| *Brachymyrmex* sp. 10 | 3 | 4 | 2 | 1 | 5 | 3 |
| *Brachymyrmex* sp. 13 | 0 | 0 | 0 | 0 | 1 | 1 |
| *Camponotus crassus* Mayr, 1862 | 21 | 4 | 0 | 5 | 0 | 0 |
| *Camponotus koseritzi* Emery, 1888 | 0 | 0 | 0 | 0 | 2 | 1 |
| *Camponotus melanoticus* Emery, 1894 | 0 | 2 | 1 | 1 | 0 | 0 |
| *Camponotus punctulatus* Mayr, 1868 | 0 | 0 | 0 | 6 | 2 | 11 |
| *Camponotus rufipes* (Fabricius, 1775) | 10 | 10 | 0 | 5 | 0 | 1 |
| *Camponotus sericeiventris* (Guérin-Méneville, 1838) | 2 | 0 | 0 | 0 | 0 | 0 |
| *Camponotus* sp. 1 | 0 | 4 | 0 | 0 | 3 | 0 |
| *Camponotus* sp. 2 | 0 | 0 | 0 | 7 | 4 | 12 |
| *Camponotus* sp. 5 | 0 | 1 | 0 | 0 | 0 | 0 |
| *Nylanderia fulva* (Mayr, 1862) | 13 | 14 | 0 | 1 | 2 | 1 |
| *Nylanderia* sp. 1 | 0 | 1 | 4 | 1 | 6 | 4 |
| **Myrmicinae** | | | | | | |
| *Acromyrmex ambiguus* (Emery, 1888) | 0 | 0 | 0 | 0 | 0 | 1 |
| *Acromyrmex coronatus* (Fabricius, 1804) | 0 | 1 | 0 | 0 | 0 | 0 |
| *Acromyrmex lobicornis* (Emery, 1888) | 0 | 0 | 0 | 2 | 0 | 0 |
| *Acromyrmex subterraneus* (Forel, 1893) | 4 | 3 | 2 | 0 | 2 | 2 |
| *Atta sexdens* (Linnaeus, 1758) | 1 | 1 | 0 | 0 | 0 | 0 |
| *Crematogaster arata** Emery, 1906 | 2 | 0 | 0 | 0 | 0 | 0 |
| *Crematogaster corticicola* Mayr, 1887 | 1 | 0 | 0 | 0 | 6 | 2 |
| *Crematogaster curvispinosa** Mayr, 1862 | 0 | 1 | 0 | 0 | 0 | 0 |
| *Crematogaster lutzi** Forel, 1905 | 0 | 0 | 2 | 0 | 0 | 0 |
| *Crematogaster quadriformis* Roger, 1863 | 1 | 10 | 0 | 3 | 1 | 9 |
| *Crematogaster* sp. 1 | 0 | 2 | 0 | 0 | 0 | 1 |

| | | | | | | |
|---|---|---|---|---|---|---|
| *Crematogaster* sp. 2 | 0 | 1 | 0 | 0 | 0 | 2 |
| *Crematogaster* sp. 5 | 1 | 0 | 0 | 0 | 0 | 0 |
| *Crematogaster* sp. 6 | 0 | 1 | 0 | 0 | 0 | 0 |
| *Ochetomyrmex semipolitus* Mayr, 1878 | 1 | 0 | 0 | 0 | 0 | 0 |
| *Pheidole* gr. *tristis* sp. 1 | 6 | 0 | 0 | 2 | 15 | 10 |
| *Pheidole* gr. *tristis* sp. 3 | 1 | 3 | 0 | 0 | 0 | 0 |
| *Pheidole aberrans* Mayr, 1868 | 0 | 0 | 0 | 0 | 0 | 1 |
| *Pheidole breviseta* Santschi, 1919 | 3 | 10 | 8 | 2 | 7 | 2 |
| *Pheidole* nr. *brunnescens* Santschi, 1929 | 2 | 4 | 0 | 0 | 0 | 0 |
| *Pheidole guilelmimuelleri** Forel, 1886 | 0 | 0 | 0 | 0 | 1 | 0 |
| *Pheidole hetschkoi* Emery, 1896 | 0 | 0 | 1 | 0 | 1 | 0 |
| *Pheidole* nr. *jelskii* Mayr, 1884 | 0 | 0 | 0 | 3 | 0 | 4 |
| *Pheidole obscurithorax* Naves, 1985 | 0 | 1 | 0 | 0 | 0 | 0 |
| *Pheidole obtusopilosa* Mayr, 1887 | 1 | 3 | 1 | 3 | 2 | 11 |
| *Pheidole pampana* Santschi, 1929 | 2 | 2 | 5 | 7 | 5 | 5 |
| *Pheidole* nr. *pubiventris* Mayr, 1887 | 7 | 0 | 28 | 2 | 19 | 9 |
| *Pheidole radoszkowskii* Mayr, 1884 | 25 | 14 | 0 | 2 | 10 | 15 |
| *Pheidole risii* Forel, 1892 | 0 | 0 | 0 | 0 | 4 | 2 |
| *Pheidole spininods* Mayr, 1887 | 0 | 0 | 0 | 0 | 0 | 2 |
| *Pheidole* sp. 3 | 4 | 4 | 0 | 0 | 4 | 6 |
| *Pheidole* sp. 9 | 0 | 0 | 0 | 1 | 0 | 0 |
| *Pheidole* sp. 13 | 0 | 0 | 0 | 0 | 1 | 0 |
| *Solenopsis invicta* Buren, 1972 | 3 | 15 | 1 | 8 | 2 | 6 |
| *Solenopsis* sp. 11 | 0 | 1 | 0 | 7 | 0 | 7 |
| *Solenopsis* sp. 14 | 0 | 1 | 6 | 4 | 13 | 6 |
| *Solenopsis* sp. 15 | 0 | 0 | 0 | 0 | 3 | 0 |
| *Solenopsis* sp. 16 | 1 | 0 | 0 | 0 | 0 | 0 |
| *Strumigenys louisianae* Roger, 1863 | 1 | 0 | 0 | 0 | 0 | 0 |
| *Trachymyrmex pruinosus* (Emery, 1906) | 0 | 0 | 0 | 1 | 0 | 0 |
| *Trachymyrmex* sp. 1 | 0 | 0 | 0 | 0 | 1 | 0 |
| *Trachymyrmex* sp. 2 | 0 | 0 | 0 | 0 | 2 | 0 |
| *Wasmannia auropunctata* (Roger, 1863) | 2 | 3 | 0 | 0 | 1 | 4 |
| *Wasmannia* sp. 1 | 0 | 0 | 1 | 6 | 2 | 1 |
| *Wasmannia* sp. 2 | 1 | 0 | 0 | 0 | 0 | 0 |
| *Wasmannia* sp. 3 | 0 | 0 | 1 | 0 | 0 | 0 |
| **Ponerinae** | | | | | | |
| *Anochetus altisquamis* Mayr, 1887 | 0 | 0 | 1 | 0 | 0 | 0 |
| *Hypoponera* sp. 11 | 0 | 0 | 0 | 1 | 0 | 0 |
| *Hypoponera* sp. 12 | 0 | 0 | 3 | 0 | 0 | 0 |
| *Neoponera crenata* (Roger, 1861) | 0 | 0 | 2 | 0 | 0 | 0 |
| *Odontomachus chelifer* (Latreille, 1802) | 0 | 0 | 0 | 0 | 1 | 0 |
| *Pachycondyla striata* Smith, 1858 | 8 | 0 | 1 | 1 | 11 | 1 |
| **Pseudomyrmecinae** | | | | | | |
| *Pseudomyrmex* nr. *flavidulus* (Smith, 1858) | 0 | 0 | 0 | 1 | 0 | 1 |
| *Pseudomyrmex gracilis* (Fabricius, 1804) | 1 | 0 | 0 | 0 | 0 | 0 |
| *Pseudomyrmex termitarius* (Smith, 1855) | 0 | 0 | 0 | 1 | 0 | 0 |
| *Pseudomyrmex* sp. 1 | 5 | 1 | 0 | 0 | 2 | 1 |
| **Total species** | **48 species** | | **40 species** | | **53 species** | |
