# Supplementary material for: Local and regional drivers of ant communities in forest-grassland ecotones in South Brazil: A taxonomic and phylogenetic approach
Source: PLoS One. 2019 Apr 11;14(4):e0215310. doi: 10.1371/journal.pone.0215310 (PMC6459495; doi:10.1371/journal.pone.0215310)
Supplement: S1 Table — COP-Canopy Openness (%); LIT-Litter Depth (cm); MOF-Air Moisture of Forests (%); MTF-Soil Surface Air Mean Temperature of Forests (°C). (PDF) [file pone.0215310.s003.pdf]

S1 Table. Local environmental variables sampled in forests from forest-grassland ecotones in Rio Grande do Sul state, Brazil. COP-Canopy Openness (%); LIT-Litter Depth (cm); MOF-Air Moisture of Forests (%); MTF-Soil Surface Air Mean Temperature of Forests (ºC).

Physiographic region	Sites	Ecotone	COP	LIT	MOF	MTF
Campanha	Santana do Livramento	A	14.24	2.06	68.42	23.52
		B	11.87	1.67	59.05	26.10
	Santo Antônio das Missões	A	13.00	2.12	69.10	27.22
		B	11.57	1.68	67.49	27.24
	São Francisco de Assis	A	9.90	4.43	78.28	20.81
		B	9.22	0.81	63.63	24.43
Campos de Cima da Serra	Cambará do Sul	A	14.29	4.68	86.23	19.13
		B	22.60	2.06	88.93	17.09
	Jaquirana	A	10.79	1.62	76.27	21.44
		B	10.77	2.12	58.50	26.65
	São Francisco de Paula	A	-	-	-	-
		B	8.44	5.68	77.86	18.42
Serra do Sudeste	Encruzilhada do Sul	A	11.96	1.68	60.76	29.33
		B	10.38	2.00	65.23	28.55
	Herval	A	15.83	2.37	71.59	26.51
		B	12.29	2.75	71.18	23.56
	Santana da Boa Vista	A	12.60	1.62	63.61	28.82
		B	30.09	2.31	55.05	32.69
