# Supplementary material for: Local and regional drivers of ant communities in forest-grassland ecotones in South Brazil: A taxonomic and phylogenetic approach
Source: PLoS One. 2019 Apr 11;14(4):e0215310. doi: 10.1371/journal.pone.0215310 (PMC6459495; doi:10.1371/journal.pone.0215310)
Supplement: S2 Table — HVE-Herbaceous Vegetation Height (cm); MOG-Air Moisture of Grasslands (%); MTG-Soil Surface Air Mean Temperature of Grasslands (°C); SHD-Shrub Density; TRD-Tree Density. (PDF) [file pone.0215310.s004.pdf]

S2 Table. Local environmental variables sampled in grasslands from forest-grassland ecotones in Rio Grande do Sul state, Brazil. HVE-Herbaceous Vegetation Height (cm); MOG-Air Moisture of Grasslands (%); MTG-Soil Surface Air Mean Temperature of Grasslands (ºC); SHD-Shrub Density; TRD-Tree Density.

Physiographic region	Sites	Ecotone	HVE	MOG	MTG	SHD	TRD
Campanha	Santana do Livramento	A	24.23	60.23	26.98	39.37	0
		B	14.33	51.33	36.05	7.87	11.87
	Santo Antônio das Missões	A	20.66	50.67	36.17	3.37	5.87
		B	30.76	61.65	34.96	6.37	13.25
	São Francisco de Assis	A	14.86	58.80	26.78	0	0.87
		B	21.73	50.99	31.91	1.37	0
Campos de Cima da Serra	Cambará do Sul	A	32.26	53.54	28.16	24.62	0
		B	11.06	79.23	18.64	7.87	18.75
	Jaquirana	A	18.23	74.14	20.45	2.50	0
		B	42.00	45.28	34.04	0	2.87
	São Francisco de Paula	A	-	-	-	-	-
		B	59.86	56.55	29.52	8.37	0
Serra do Sudeste	Encruzilhada do Sul	A	6.50	59.31	32.57	4.75	0.25
		B	8.56	65.83	28.49	1.25	9.87
	Herval	A	32.40	64.88	30.31	28.87	5.62
		B	23.90	65.56	27.17	24.12	15.62
	Santana da Boa Vista	A	18.36	62.82	30.36	10.12	3.62
		B	8.33	51.32	35.84	4.75	5.62
