# Supplementary material for: Local and regional drivers of ant communities in forest-grassland ecotones in South Brazil: A taxonomic and phylogenetic approach
Source: PLoS One. 2019 Apr 11;14(4):e0215310. doi: 10.1371/journal.pone.0215310 (PMC6459495; doi:10.1371/journal.pone.0215310)
Supplement: S3 Table — V1-Annual Mean Temperature (°C); V2-Temperature Seasonality (°C); V3-Minimum Temperature of Coldest Month (°C); V4-Annual Precipitation (mm); V5-Precipitation Seasonality (%); V6-Mean Altitude (m). (PDF) [file pone.0215310.s005.pdf]

S3 Table. Regional variables obtained from WorldClim (V1 to V5) and Shuttle Radar Topographic Mission (V6) to three different physiographic regions from Rio Grande do Sul state, Brazil. V1-Annual Mean Temperature (ºC); V2-Temperature Seasonality (ºC); V3-Minimum Temperature of Coldest Month (ºC); V4-Annual Precipitation (mm); V5-Precipitation Seasonality (%); V6-Mean Altitude (m).

Physiographic region	Sites	V1	V2	V3	V4	V5	V6
Campanha	Santana do Livramento	18.5	443.5	6.9	1588	10	279
	Santo Antônio das Missões	21.1	393.9	9.8	1907	12	113
	São Francisco de Assis	19.0	407.9	8.1	1876	9	162
Campos de Cima da Serra	Cambará do Sul	14.6	286.1	6.3	1876	10	979
	Jaquirana	16.0	291.0	7.2	2048	9	836
	São Francisco de Paula	14.9	301.6	6.6	1957	8	835
Serra do Sudeste	Encruzilhada do Sul	18.1	345.8	8.9	1616	10	382
	Herval	17.4	389.8	6.7	1340	12	244
	Santana da Boa Vista	19.6	360.3	10.1	1577	12	93
