# Supplementary material for: Local and regional drivers of ant communities in forest-grassland ecotones in South Brazil: A taxonomic and phylogenetic approach
Source: PLoS One. 2019 Apr 11;14(4):e0215310. doi: 10.1371/journal.pone.0215310 (PMC6459495; doi:10.1371/journal.pone.0215310)
Supplement: S4 Table — Variance inflation factor (VIF) table with local environmental variables sampled in (a) forests and (b) grasslands from forest-grassland ecotones in Rio Grande do Sul state, Brazil. Bold numbers means multicollinearity between variables (VIF > 3). COP-Canopy Openness (%); LIT-Litter Depth (cm); MOF-Air Moisture of Forests (%); MTF-Soil Surface Air Mean Temperature of Forests (°C); HVE-Herbaceous Vegetation Height (cm); MOG-Air Moisture of Grasslands (%); MTG-Soil Surface Air Mean Temperature of Grasslands (°C); SHD-Shrub Density; TRD-Tree Density. (PDF) [file pone.0215310.s006.pdf]

S4 Table. Variance inflation factor (VIF) table with local environmental variables sampled in (a) forests and (b) grasslands from forest-grassland ecotones in Rio Grande do Sul state, Brazil. Bold numbers means multicollinearity between variables (VIF > 3). COP-Canopy Openness (%); LIT-Litter Depth (cm); MOF-Air Moisture of Forests (%); MTF-Soil Surface Air Mean Temperature of Forests (ºC); HVE-Herbaceous Vegetation Height (cm); MOG-Air Moisture of Grasslands (%); MTG-Soil Surface Air Mean Temperature of Grasslands (ºC); SHD-Shrub Density; TRD-Tree Density.

(a)

	COP	LIT	MOF	MTF
COP				
LIT	1.012			
MOF	1.001	1.439		
MTF	1.053	1.445	**4.683**	

(b)

	HVE	MOG	MTG	SHD	TRD
HVE					
MOG	1.085				
MTG	1.009	**3.039**			
SHD	1.051	1.024	1.038		
TRD	1.074	1.201	1.008	1.000	
