# Supplementary material for: Local and regional drivers of ant communities in forest-grassland ecotones in South Brazil: A taxonomic and phylogenetic approach
Source: PLoS One. 2019 Apr 11;14(4):e0215310. doi: 10.1371/journal.pone.0215310 (PMC6459495; doi:10.1371/journal.pone.0215310)
Supplement: S5 Table — Bold numbers means multicollinearity between variables (VIF > 3). V1-Annual Mean Temperature (°C); V2-Temperature Seasonality (°C); V3-Minimum Temperature of Coldest Month (°C); V4-Annual Precipitation (mm); V5-Precipitation Seasonality (%); V6-Mean Altitude (m). (PDF) [file pone.0215310.s007.pdf]

S5 Table. Variance inflation factor (VIF) table with regional variables obtained to three different physiographic regions from Rio Grande do Sul state, Brazil. Bold numbers means multicollinearity between variables (VIF > 3). V1-Annual Mean Temperature (ºC); V2-Temperature Seasonality (ºC); V3-Minimum Temperature of Coldest Month (ºC); V4-Annual Precipitation (mm); V5-Precipitation Seasonality (%); V6-Mean Altitude (m).

	V1	V2	V3	V4	V5	V6
V1						
V2	2.341					
V3	**3.052**	1.087				
V4	1.091	1.378	1.006			
V5	1.632	1.199	1.385	1.617		
V6	**7.507**	**3.642**	1.881	1.432	1.686	
