# Supplementary material for: Local and regional drivers of ant communities in forest-grassland ecotones in South Brazil: A taxonomic and phylogenetic approach
Source: PLoS One. 2019 Apr 11;14(4):e0215310. doi: 10.1371/journal.pone.0215310 (PMC6459495; doi:10.1371/journal.pone.0215310)
Supplement: S6 Table — (PDF) [file pone.0215310.s008.pdf]

S6 Table. Geographical coordinates obtained from one central point between ecotones in each site.

Physiographic region	Sites	Latitude	Longitude
Campanha	Santana do Livramento	-30.719103	-55.512003
	Santo Antônio das Missões	-28.530828	-55.423558
	São Francisco de Assis	-29.628075	-55.137556
Campos de Cima da Serra	Cambará do Sul	-29.164836	-50.057197
	Jaquirana	-29.016753	-50.395103
	São Francisco de Paula	-29.483322	-50.204753
Serra do Sudeste	Encruzilhada do Sul	-30.561036	-52.562158
	Herval	-32.093058	-53.621503
	Santana da Boa Vista	-30.932525	-53.023883
